# Redetermination of l-tryptophan hydro­bromide

**DOI:** 10.1107/S1600536809017322

**Published:** 2009-05-14

**Authors:** Kirsty Stewart

**Affiliations:** a School of Chemical and Physical Sciences, University of KwaZulu-Natal, Scottsville 3209, South Africa

## Abstract

The redetermined crystal structure of the title compound, C_11_H_13_N_2_O_2_^+^·Br^−^, is reported. Data collection at 100 K about three crystallographic axes resulted in a crystal structure with significantly higher precision in comparison to the two-dimensional data collected at 176 K  [Takigawa *et al.* [(1966) *Bull. Chem. Soc. Jpn*, **39**, 2369–2378]. The carboxyl group and indole ring system are planar, with maximum deviations of 0.002 (2) and 0.007 (2) Å, respectively, and make an angle of 70.17 (1)° with each other. The mol­ecules are arranged in double layers of carboxyl and amino groups parallel to the *ab* plane, stabilized by an extensive network of N—H⋯Br and O—H⋯Br hydrogen bonds. The polar layer is held together by a network of three N—H⋯Br hydrogen bonds and one O—H⋯Br hydrogen bond. In the non-polar layer, the indole rings are linked mainly by electrostatic N—H⋯C inter­actions between the polarized bond N—H (H is δ^+^) of the pyrrole unit and two of the ring C atoms (δ^−^) of the benzene rings of adjacent mol­ecules. The distances of these electrostatic inter­actions are 2.57 and 2.68 Å, respectively. C—H⋯O and C—H⋯π inter­actions are also present.

## Related literature

For a previous determination of the crystal structure of the title compound, see: Takigawa *et al.* (1966 ▶). Study of crystal structures of amino acids and their complexes has provided information about aggregation and the effect of other mol­ecules on their inter­actions and mol­ecular properties, see: Vijayan (1988 ▶); Prasad & Vijayan (1993 ▶). For the structure of histidine hydro­chloride monohydrate, see: Takigawa *et al.* (1966 ▶). Donohue & Caron (1964 ▶). The structures of many amino acids with non-polar side chains feature a double-layered arrangement, see: Harding & Long (1968 ▶); Torii & Iitaka (1970 ▶, 1971 ▶, 1973 ▶).
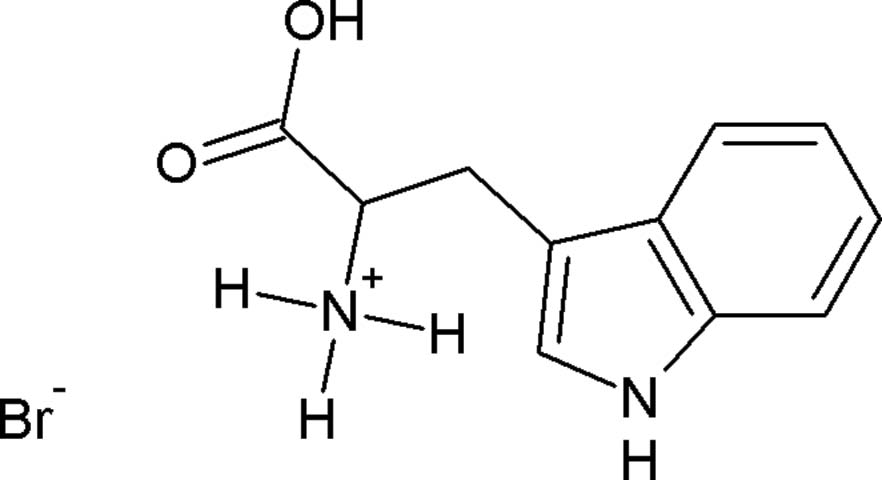


## Experimental

### 

#### Crystal data

C_11_H_13_N_2_O_2_^+^·Br^−^
*M* *_r_* = 285.13
Monoclinic, 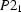

*a* = 7.6272 (3) Å
*b* = 5.3840 (2) Å
*c* = 14.4358 (5) Å
β = 100.688 (3)°
*V* = 582.52 (4) Å^3^
*Z* = 2
Mo *K*α radiation
μ = 3.52 mm^−1^
*T* = 100 K
0.40 × 0.15 × 0.15 mm

#### Data collection

Oxford Xcalibur2 CCD diffractometer
Absorption correction: multi-scan (SCALE3 ABSPACK in *CrysAlis RED*; Oxford Diffraction, 2008 ▶) *T*_min_ = 0.334, *T*_max_ = 0.621
5749 measured reflections
2731 independent reflections
2507 reflections with *I* > 2σ(*I*)
*R*_int_ = 0.024

#### Refinement

*R*[*F* ^2^ > 2σ(*F* ^2^)] = 0.027
*wR*(*F* ^2^) = 0.063
*S* = 1.01
2731 reflections
147 parameters
1 restraint
H-atom parameters constrained
Δρ_max_ = 0.33 e Å^−3^
Δρ_min_ = −1.14 e Å^−3^
Absolute structure: Flack (1983 ▶), 523 Freidel pairs
Flack parameter: 0.009 (9)

### 

Data collection: *CrysAlis CCD* (Oxford Diffraction, 2008 ▶); cell refinement: *CrysAlis RED* (Oxford Diffraction, 2008 ▶); data reduction: *CrysAlis RED*; program(s) used to solve structure: *SHELXS97* (Sheldrick, 2008 ▶); program(s) used to refine structure: *SHELXL97* (Sheldrick, 2008 ▶); molecular graphics: *ORTEP-3* (Farrugia, 1997 ▶); software used to prepare material for publication: *WinGX* (Farrugia, 1999 ▶).

## Supplementary Material

Crystal structure: contains datablocks I, global. DOI: 10.1107/S1600536809017322/at2781sup1.cif

Structure factors: contains datablocks I. DOI: 10.1107/S1600536809017322/at2781Isup2.hkl

Additional supplementary materials:  crystallographic information; 3D view; checkCIF report

## Figures and Tables

**Table 1:** Hydrogen-bond geometry (Å, °)

*D*—H⋯*A*	*D*—H	H⋯*A*	*D*⋯*A*	*D*—H⋯*A*
C7—H4⋯*Cg*1^i^	0.95	2.66	3.494 (3)	146
N1—H5⋯*Cg*2^i^	0.88	2.72	3.406 (2)	136
N2—H11⋯Br1^ii^	0.91	2.56	3.3208 (17)	142
N2—H12⋯Br1^iii^	0.91	2.42	3.322 (3)	173
N2—H13⋯Br1^iv^	0.91	2.52	3.320 (3)	147
C4—H1⋯Br1^iii^	0.95	2.85	3.750 (2)	159
C10—H9⋯O1^v^	1.00	2.49	3.404 (3)	153
C10—H9⋯O1^ii^	1.00	2.56	3.199 (3)	121
O2—H10⋯Br1	0.84	2.34	3.173 (2)	169

Symmetry codes: (i) 
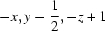
; (ii) 
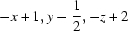
; (iii) 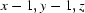; (iv) 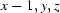; (v) 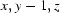. *Cg*1 is the centroid of the N1/C1–C3/C8 ring and *Cg*2 is the centroid of the C3–C8 ring.

**Fig. 1.**
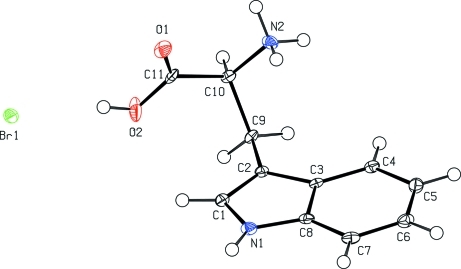
Molecular structure of (L)-2-amino-3-(1H-indol-3-yl)-propionic acid hydrobromide, showing 50% probability displacement ellipsoids and atomic numbering.

**Fig. 2.**
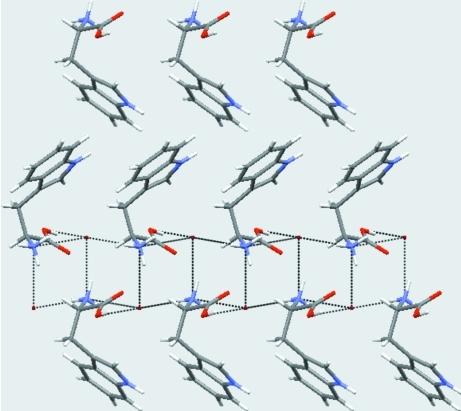
Packing diagram showing a view along the a axis.

## supplementary crystallographic information

### Comment

The crystal structures of amino acids and their complexes have provided interesting information about aggregation, and the effect of other molecules on their interaction and molecular properties (Vijayan, 1988; Prasad & Vijayan 1993).

The crystal structure of *L*-tryptophan hydrobromide was determined some 40 years ago (Takigawa *et al.*, 1966). The final refinement was carried out to only *R* = 0.101 with no data available for H atoms. The reported structure possesses almost identical crystal parameters to the structure reported here in terms of space group and unit-cell dimensions and angles. However, the collection of data at 100 K about three crystallographic axes in comparison to the data reported by Takigawa *et al.* resulted in more accurate data thus allowing some previously unidentified notable conclusions to be drawn about the crystal structure.

The C—N distance 1.488 (3) Å coincides well with those in *e.g.* histidine hydrochloride monohydrate (1.495 Å) (Donohue & Caron, 1964). The two C—O bond lengths are 1.316 (3) Å and 1.204 (3) Å for C11—O2 and C11—O1, respectively and the three angles around the C11 atom are O2—C11—O1 = 125.8 (2)°, O2—C11—C10 = 110.0 (2) ° and O1—C11—C10 = 124.1 (2) °.

The planarity of the carboxyl group with the α-carbon has been established in many investigations and the deviations of the amino nitrogen range from 0.00 to 0.82 Å for the amino acids so far investigated. For the present molecule, the amino nitrogen is 0.094 Å out of the plane, so the amide group is essentially planar in this case.

The mean plane through the atoms of the indole ring with the methylene carbon attached to it forms a dihedral angle of 70.17 (1) ° with the mean plane of the carboxyl group.

The structures of many amino acids with non-polar side chains have the arrangement of a double layered system (Torii & Iitaka 1970; Torii & Iitaka, 1971; Torii & Iitaka, 1973; Harding & Long, 1968) which is characteristic for a structure containing polar and non-polar groups together. The polar layer is held together by a network of hydrogen bonds between the halide ions and the amino nitrogen and the halide ions and the carboxyl group.

The amino nitrogen forms three N—H···Br hydrogen bonds, in the lengths of 2.56 (1) Å, 2.41 (1) Å and 2.52 (1) Å. The three acceptor halogen ions are approximately at the three vertices of a regular tetrahedron centred around the nitrogen atom, with the fourth vortex positioned in the direction of the α-carbon.

The fourth hydrogen bond completing the network is a O—H···Br^-^ which is 2.34 (1) Å in length.

In the non-polar layer, the indole rings are packed in a manner similar to that found for typical aromatic molecules. A weak electrostatic interaction with a separation of 2.677 (1) Å exists between N1—H5 from the pyrrole moiety and the slightly positively charged C6 from the benzene moiety of a neighbouring symmetry related molecule [-*x*, -1/2 + *y*, 1 - *z*]. A similar interaction with a separation of 2.573 (1) Å exists between the same N1—H5 and C7 from the benzene moiety of molecule [-*x*, -1/2 + *y*, 1 - *z*]. The metrics of such interactions are reflected in the T-shaped edge-to-face geometry in the non-polar layer.

### Experimental

*L*-tryptophan hydrobromide was obtained in the form of plate-like crystals by dissolving *L*-tryptophan in concentrated hydrobromic acid followed by slow evaporation at room temperature.

HRMS (ES^-^): Found [M—H] 283.0082 C_11_H_12_N_2_O_2_Br Expected 283.0080 (-0.7 ppm).

### Refinement

All H atoms were positioned in geometrically idealized positions and constrained to ride on their parent atoms with N—H = 0.91 Å, O—H = 0.84 Å and C—H distances in the range 0.95–1.00 Å. and *U*_iso_(H)= 1.2–1.5 *U*_eq_(parent atom).

### Crystal data

**Table:**

C_11_H_13_N_2_O_2_^+^·Br^−^	*F*(000) = 288
*M**_r_* = 285.13	*D*_x_ = 1.626 Mg m^−^^3^
Monoclinic, *P*2_1_	Mo *K*α radiation, λ = 0.71073 Å
Hall symbol: P 2yb	Cell parameters from 4434 reflections
*a* = 7.6272 (3) Å	θ = 3.8–31.9°
*b* = 5.3840 (2) Å	µ = 3.52 mm^−^^1^
*c* = 14.4358 (5) Å	*T* = 100 K
β = 100.688 (3)°	Needle, colourless
*V* = 582.52 (4) Å^3^	0.40 × 0.15 × 0.15 mm
*Z* = 2	

### Data collection

**Table:**

Oxford Xcalibur2 CCD diffractometer	2731 independent reflections
Radiation source: Enhance (Mo) X-ray Source	2507 reflections with *I* > 2σ(*I*)
graphite	*R*_int_ = 0.024
Detector resolution: 8.4190 pixels mm^-1^	θ_max_ = 32.0°, θ_min_ = 4.1°
ω/2θ scans	*h* = −11→10
Absorption correction: multi-scan (SCALE3 ABSPACK in *CrysAlis RED*; Oxford Diffraction, 2008)	*k* = −6→7
*T*_min_ = 0.334, *T*_max_ = 0.621	*l* = −21→21
5749 measured reflections	

### Refinement

**Table:**

Refinement on *F*^2^	Secondary atom site location: difference Fourier map
Least-squares matrix: full	Hydrogen site location: inferred from neighbouring sites
*R*[*F*^2^ > 2σ(*F*^2^)] = 0.027	H-atom parameters constrained
*wR*(*F*^2^) = 0.063	*w* = 1/[σ^2^(*F*_o_^2^) + (0.0388*P*)^2^] where *P* = (*F*_o_^2^ + 2*F*_c_^2^)/3
*S* = 1.01	(Δ/σ)_max_ < 0.001
2731 reflections	Δρ_max_ = 0.33 e Å^−^^3^
147 parameters	Δρ_min_ = −1.14 e Å^−^^3^
1 restraint	Absolute structure: Flack (1983), 523 Freidel pairs
Primary atom site location: structure-invariant direct methods	Flack parameter: 0.009 (9)

### Special details

**Table:**

Geometry. All e.s.d.'s (except the e.s.d. in the dihedral angle between two l.s. planes) are estimated using the full covariance matrix. The cell e.s.d.'s are taken into account individually in the estimation of e.s.d.'s in distances, angles and torsion angles; correlations between e.s.d.'s in cell parameters are only used when they are defined by crystal symmetry. An approximate (isotropic) treatment of cell e.s.d.'s is used for estimating e.s.d.'s involving l.s. planes.
Refinement. Refinement of *F*^2^ against ALL reflections. The weighted *R*-factor *wR* and goodness of fit *S* are based on *F*^2^, conventional *R*-factors *R* are based on *F*, with *F* set to zero for negative *F*^2^. The threshold expression of *F*^2^ > σ(*F*^2^) is used only for calculating *R*-factors(gt) *etc*. and is not relevant to the choice of reflections for refinement. *R*-factors based on *F*^2^ are statistically about twice as large as those based on *F*, and *R*- factors based on ALL data will be even larger.

### Fractional atomic coordinates and isotropic or equivalent isotropic displacement parameters (Å^2^)

**Table:**

	*x*	*y*	*z*	*U*_iso_*/*U*_eq_	
C1	0.2755 (3)	0.4954 (5)	0.66336 (17)	0.0112 (4)	
H6	0.4001	0.5280	0.6774	0.013*	
C2	0.1920 (3)	0.3004 (4)	0.69909 (15)	0.0095 (5)	
C3	0.0055 (3)	0.3213 (4)	0.65929 (15)	0.0086 (4)	
C4	−0.1449 (3)	0.1811 (5)	0.66802 (15)	0.0111 (4)	
H1	−0.1339	0.0367	0.7065	0.013*	
C5	−0.3108 (3)	0.2569 (9)	0.61935 (14)	0.0141 (4)	
H2	−0.4140	0.1638	0.6253	0.017*	
C6	−0.3286 (3)	0.4686 (5)	0.56162 (17)	0.0141 (5)	
H3	−0.4436	0.5159	0.5291	0.017*	
C7	−0.1815 (3)	0.6103 (5)	0.55101 (17)	0.0125 (4)	
H4	−0.1932	0.7530	0.5116	0.015*	
C8	−0.0151 (3)	0.5338 (5)	0.60080 (16)	0.0103 (4)	
C9	0.2770 (3)	0.0989 (5)	0.76424 (16)	0.0100 (4)	
H7	0.3862	0.0421	0.7428	0.012*	
H8	0.1939	−0.0438	0.7591	0.012*	
C10	0.3269 (3)	0.1746 (5)	0.86847 (15)	0.0109 (4)	
H9	0.3832	0.0280	0.9049	0.013*	
C11	0.4583 (3)	0.3889 (5)	0.88531 (16)	0.0124 (5)	
N1	0.1513 (3)	0.6363 (4)	0.60405 (14)	0.0120 (4)	
H5	0.1746	0.7698	0.5733	0.014*	
N2	0.1651 (2)	0.2443 (6)	0.90642 (12)	0.0142 (3)	
H13	0.1107	0.3761	0.8735	0.021*	
H11	0.1972	0.2860	0.9683	0.021*	
H12	0.0885	0.1133	0.9006	0.021*	
O1	0.4357 (3)	0.5748 (4)	0.92793 (13)	0.0166 (4)	
O2	0.5974 (2)	0.3408 (4)	0.84628 (15)	0.0229 (4)	
H10	0.6731	0.4547	0.8596	0.034*	
Br1	0.90610 (3)	0.74408 (5)	0.873511 (13)	0.01394 (7)	

### Atomic displacement parameters (Å^2^)

**Table:**

	*U*^11^	*U*^22^	*U*^33^	*U*^12^	*U*^13^	*U*^23^
C1	0.0119 (10)	0.0122 (12)	0.0092 (10)	−0.0014 (9)	0.0012 (8)	−0.0008 (8)
C2	0.0108 (9)	0.0105 (14)	0.0072 (9)	0.0005 (7)	0.0017 (7)	−0.0011 (7)
C3	0.0107 (10)	0.0096 (10)	0.0051 (9)	0.0013 (7)	0.0001 (7)	−0.0008 (7)
C4	0.0132 (10)	0.0116 (11)	0.0080 (9)	0.0002 (8)	0.0005 (7)	0.0008 (7)
C5	0.0117 (9)	0.0175 (11)	0.0133 (9)	−0.0010 (17)	0.0028 (7)	−0.0037 (17)
C6	0.0122 (11)	0.0190 (13)	0.0104 (11)	0.0051 (9)	0.0002 (8)	0.0005 (9)
C7	0.0176 (11)	0.0114 (11)	0.0084 (10)	0.0043 (9)	0.0024 (8)	0.0010 (8)
C8	0.0150 (11)	0.0099 (10)	0.0063 (10)	−0.0006 (9)	0.0027 (8)	−0.0012 (8)
C9	0.0107 (10)	0.0092 (10)	0.0089 (10)	−0.0004 (8)	−0.0010 (8)	−0.0020 (8)
C10	0.0142 (11)	0.0095 (10)	0.0081 (10)	−0.0010 (8)	−0.0002 (7)	−0.0002 (8)
C11	0.0128 (10)	0.0127 (12)	0.0091 (10)	−0.0035 (9)	−0.0048 (8)	0.0010 (9)
N1	0.0172 (10)	0.0092 (10)	0.0095 (9)	−0.0020 (8)	0.0023 (7)	0.0019 (7)
N2	0.0194 (8)	0.0124 (8)	0.0120 (7)	−0.0064 (15)	0.0062 (6)	−0.0017 (14)
O1	0.0216 (9)	0.0114 (9)	0.0158 (9)	−0.0046 (7)	0.0009 (7)	−0.0030 (7)
O2	0.0124 (9)	0.0217 (10)	0.0354 (11)	−0.0078 (8)	0.0061 (8)	−0.0112 (9)
Br1	0.01610 (10)	0.01382 (11)	0.01214 (9)	−0.00518 (15)	0.00322 (6)	0.00128 (14)

### Geometric parameters (Å, °)

**Table:**

C1—C2	1.377 (3)	C8—N1	1.377 (3)
C1—N1	1.381 (3)	C9—C10	1.537 (3)
C1—H6	0.9500	C9—H7	0.9900
C2—C3	1.436 (3)	C9—H8	0.9900
C2—C9	1.502 (3)	C10—N2	1.488 (3)
C3—C4	1.398 (3)	C10—C11	1.518 (3)
C3—C8	1.413 (3)	C10—H9	1.0000
C4—C5	1.390 (3)	C11—O1	1.204 (3)
C4—H1	0.9500	C11—O2	1.316 (3)
C5—C6	1.404 (5)	N1—H5	0.8800
C5—H2	0.9500	N2—H13	0.9100
C6—C7	1.389 (4)	N2—H11	0.9100
C6—H3	0.9500	N2—H12	0.9100
C7—C8	1.399 (3)	O2—H10	0.8400
C7—H4	0.9500		
			
C2—C1—N1	109.8 (2)	C2—C9—H7	108.5
C2—C1—H6	125.1	C10—C9—H7	108.5
N1—C1—H6	125.1	C2—C9—H8	108.5
C1—C2—C3	106.5 (2)	C10—C9—H8	108.5
C1—C2—C9	127.5 (2)	H7—C9—H8	107.5
C3—C2—C9	126.0 (2)	N2—C10—C11	108.5 (2)
C4—C3—C8	119.2 (2)	N2—C10—C9	110.80 (18)
C4—C3—C2	133.6 (2)	C11—C10—C9	113.26 (19)
C8—C3—C2	107.1 (2)	N2—C10—H9	108.0
C5—C4—C3	118.8 (3)	C11—C10—H9	108.0
C5—C4—H1	120.6	C9—C10—H9	108.0
C3—C4—H1	120.6	O1—C11—O2	125.8 (2)
C4—C5—C6	121.1 (3)	O1—C11—C10	124.1 (2)
C4—C5—H2	119.4	O2—C11—C10	110.0 (2)
C6—C5—H2	119.4	C8—N1—C1	108.9 (2)
C7—C6—C5	121.4 (2)	C8—N1—H5	125.6
C7—C6—H3	119.3	C1—N1—H5	125.6
C5—C6—H3	119.3	C10—N2—H13	109.5
C6—C7—C8	117.1 (2)	C10—N2—H11	109.5
C6—C7—H4	121.4	H13—N2—H11	109.5
C8—C7—H4	121.4	C10—N2—H12	109.5
N1—C8—C7	129.9 (2)	H13—N2—H12	109.5
N1—C8—C3	107.7 (2)	H11—N2—H12	109.5
C7—C8—C3	122.4 (2)	C11—O2—H10	109.5
C2—C9—C10	114.9 (2)		
			
N1—C1—C2—C3	0.3 (3)	C2—C3—C8—N1	0.6 (2)
N1—C1—C2—C9	178.4 (2)	C4—C3—C8—C7	0.1 (3)
C1—C2—C3—C4	179.8 (2)	C2—C3—C8—C7	−179.7 (2)
C9—C2—C3—C4	1.6 (4)	C1—C2—C9—C10	78.8 (3)
C1—C2—C3—C8	−0.5 (2)	C3—C2—C9—C10	−103.4 (3)
C9—C2—C3—C8	−178.7 (2)	C2—C9—C10—N2	62.4 (3)
C8—C3—C4—C5	−0.6 (3)	C2—C9—C10—C11	−59.8 (3)
C2—C3—C4—C5	179.1 (3)	N2—C10—C11—O1	4.3 (3)
C3—C4—C5—C6	0.6 (4)	C9—C10—C11—O1	127.8 (3)
C4—C5—C6—C7	−0.1 (4)	N2—C10—C11—O2	−175.31 (19)
C5—C6—C7—C8	−0.4 (4)	C9—C10—C11—O2	−51.8 (3)
C6—C7—C8—N1	−179.9 (2)	C7—C8—N1—C1	179.9 (2)
C6—C7—C8—C3	0.4 (3)	C3—C8—N1—C1	−0.4 (3)
C4—C3—C8—N1	−179.7 (2)	C2—C1—N1—C8	0.1 (3)

### Hydrogen-bond geometry (Å, °)

**Table:**

*D*—H···*A*	*D*—H	H···*A*	*D*···*A*	*D*—H···*A*
C7—H4···Cg1^i^	0.95	2.66	3.494 (3)	146
N1—H5···Cg2^i^	0.88	2.72	3.406 (2)	136
N2—H11···Br1^ii^	0.91	2.56	3.3208 (17)	142
N2—H12···Br1^iii^	0.91	2.42	3.322 (3)	173
N2—H13···Br1^iv^	0.91	2.52	3.320 (3)	147
C4—H1···Br1^iii^	0.95	2.85	3.750 (2)	159
C10—H9···O1^v^	1.00	2.49	3.404 (3)	153
C10—H9···O1^ii^	1.00	2.56	3.199 (3)	121
O2—H10···Br1	0.84	2.34	3.173 (2)	169

Symmetry codes: (i) −*x*, *y*−1/2, −*z*+1; (ii) −*x*+1, *y*−1/2, −*z*+2; (iii) *x*−1, *y*−1, *z*; (iv) *x*−1, *y*, *z*; (v) *x*, *y*−1, *z*.
